# Supplementary material for: MLL1 is required for PAX7 expression and satellite cell self-renewal in mice
Source: Nat Commun. 2019 Sep 18;10:4256. doi: 10.1038/s41467-019-12086-9 (PMC6751293; doi:10.1038/s41467-019-12086-9)
Supplement: Supplementary file 1 — Supplementary Information [file 41467_2019_12086_MOESM1_ESM.pdf]

SUPPLEMENTARY INFORMATION

MLL1 is Required for PAX7 Expression and Satellite Cell Self-Renewal in mice

Gregory C. Addicks[1,2,6], Caroline E. Brun[1,2,6], Marie-Claude Sincennes[1,2], John Saber[1,2], Christopher J. Porter[3], A. Francis Stewart[4], Patricia Ernst[5], and Michael A. Rudnicki[1,2,*]

[1] Sprott Centre for Stem Cell Research, Regenerative Medicine Program, Ottawa Hospital Research Institute, Ottawa, ON, K1H 8L6, Canada
[2] Department of Cellular and Molecular Medicine, Faculty of Medicine, University of Ottawa, Ottawa, ON, K1H 8M5, Canada
[3] Sprott Centre for Stem Cell Research, Ottawa Bioinformatics Core Facility, Ottawa Hospital Research Institute, Ottawa, ON, K1H 8L6, Canada
[4] Genomics, Center for Molecular and Cellular Bioengineering, Technische Universität Dresden, Tatzberg 47, Dresden 01307, Germany
[5] Department of Pediatrics and Pharmacology, University of Colorado/Anschutz Medical Campus, Aurora, CO 80045, USA
[6] These authors contributed equally to this work.
* email: mrudnicki@ohri.ca

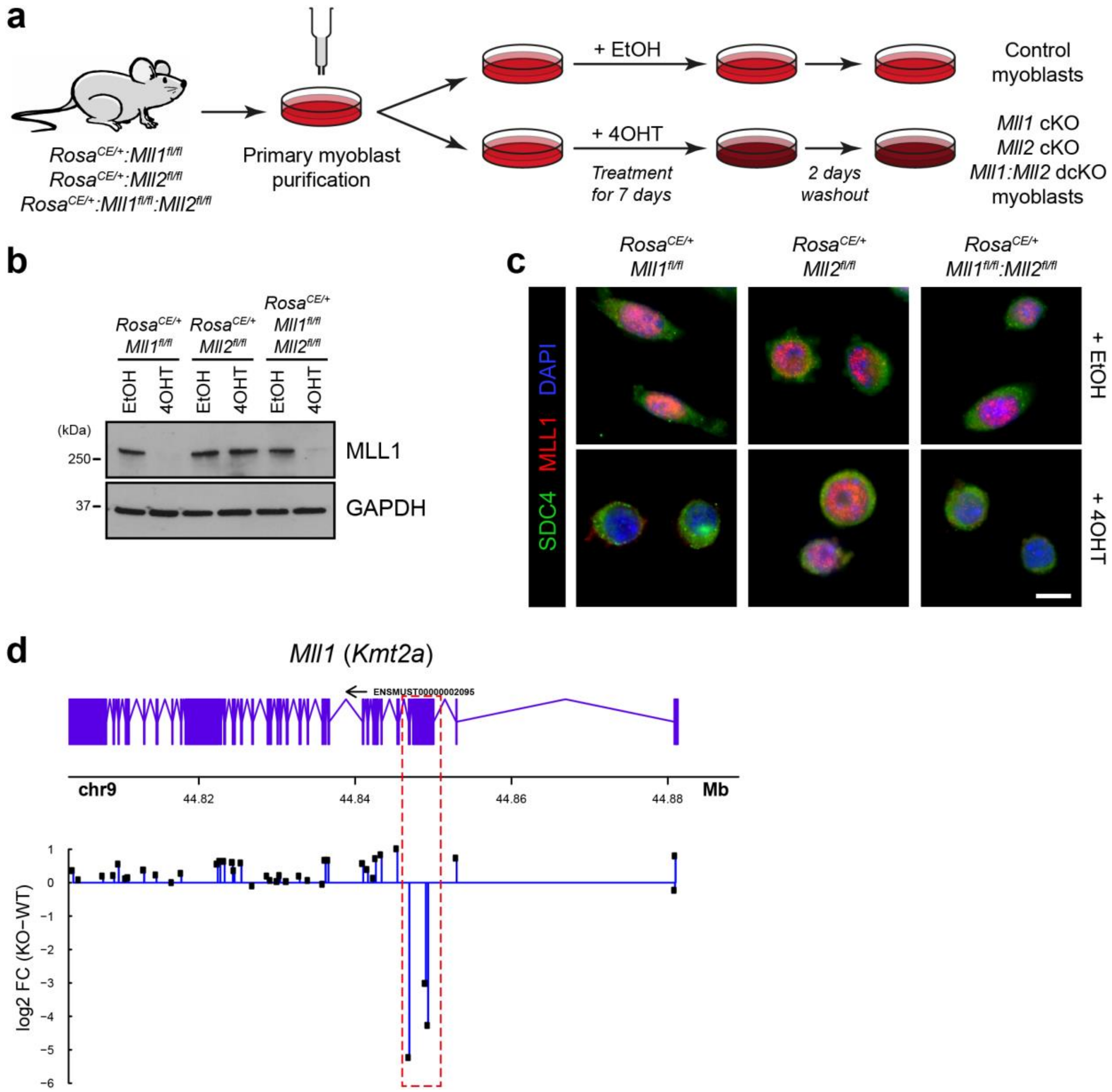

Supplementary Figure 1. Generation of *Mll1* cKO, *Mll2* cKO and *Mll1:Mll2* dcKO primary myoblasts.

(a) Experimental schematic outlining the protocol followed to obtain *Mll1* cKO, *Mll2* cKO and *Mll1:Mll2* dcKO primary myoblasts derived from *Rosa*$^{CE/+}$:*Mll1*$^{fl/fl}$, *Rosa*$^{CE/+}$:*Mll2*$^{fl/fl}$ and *Rosa*$^{CE/+}$:*Mll1*$^{fl/fl}$:*Mll2*$^{fl/fl}$ mice. To generate the cKO/dcKO myoblasts and their controls, purified primary myoblasts were cultured with either with 4-hydroxytamoxifen (4-OHT) allowing for CreER translocation to the nucleus and the Cre/LoxP-mediated recombination, or with vehicle (ethanol 96%, EtOH), respectively.

(b) Western blot analysis of MLL1 in control and *Mll1* cKO primary myoblasts. GAPDH is used as a loading control.

(c) Immunostaining of SYNDECAN-4 (SDC4, green) and MLL1 (red) in control and *Mll1* cKO, *Mll2* cKO and *Mll1:Mll2* dcKO primary myoblasts. Nuclei are counterstained with DAPI (blue). Scale bar represents 10µm.

(d) Log2 fold change values for each probe sets spanning *Mll1* transcript in *Mll1* cKO primary myoblasts. Red-dashed box indicates the exons 3 and 4 within the floxed region.

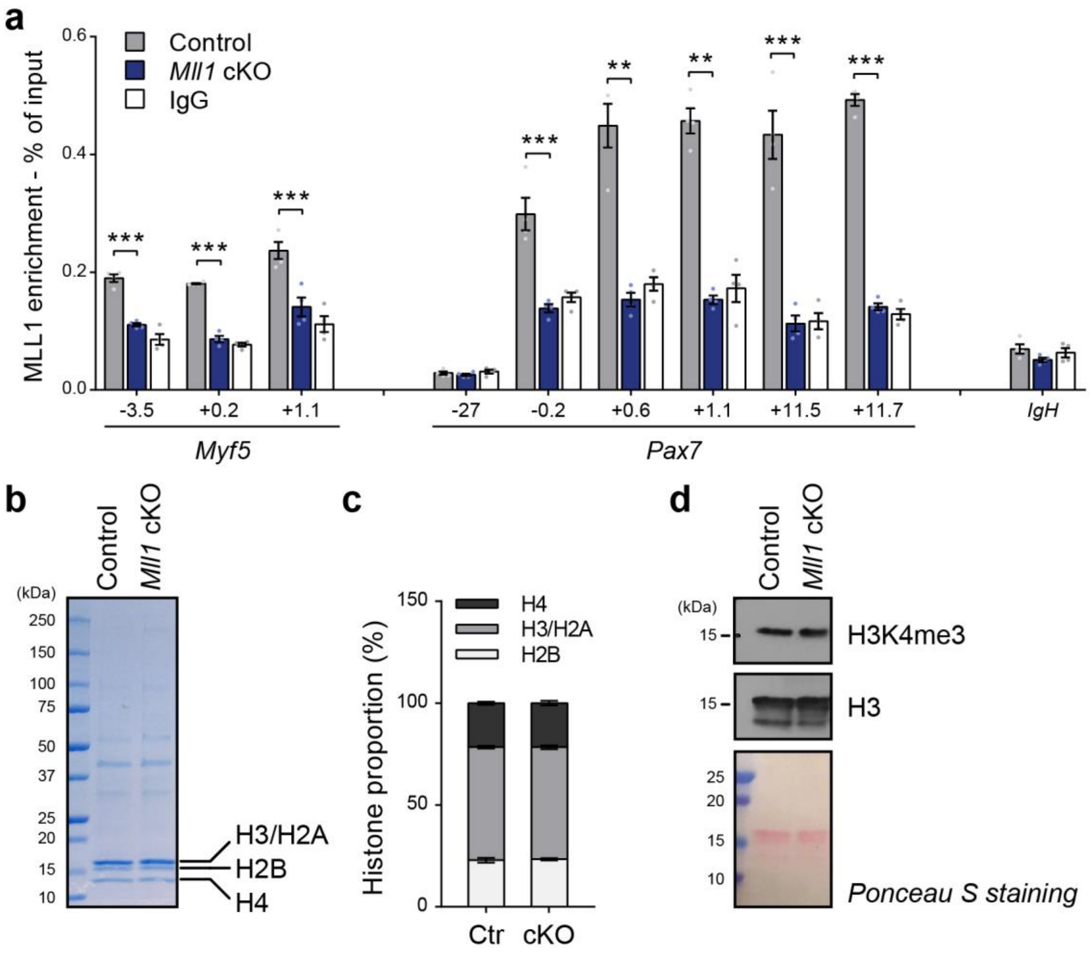

Supplementary Figure 2. Loss of MLL1 does not change the global level of H3K4me3.
(a) ChIP-qPCR analysis of MLL1 recruitment to *Pax7* and *Myf5* loci in control and *Mll1* cKO myoblasts. Data are presented as the mean ± S.E.M. of 4 independent experiments.
(b) Coomassie blue of total histone extracts from control and *Mll1* cKO myoblasts.
(c) H3/H2A, H2B and H4 histone proportions of control and *Mll1* cKO myoblasts. Data are presented as the mean ± S.E.M. of 4 biological replicates.
(d) Western blot analysis of H3K4me3 and total H3 in control and *Mll1* cKO primary myoblasts. The Ponceau S staining ensures the equivalent loading.

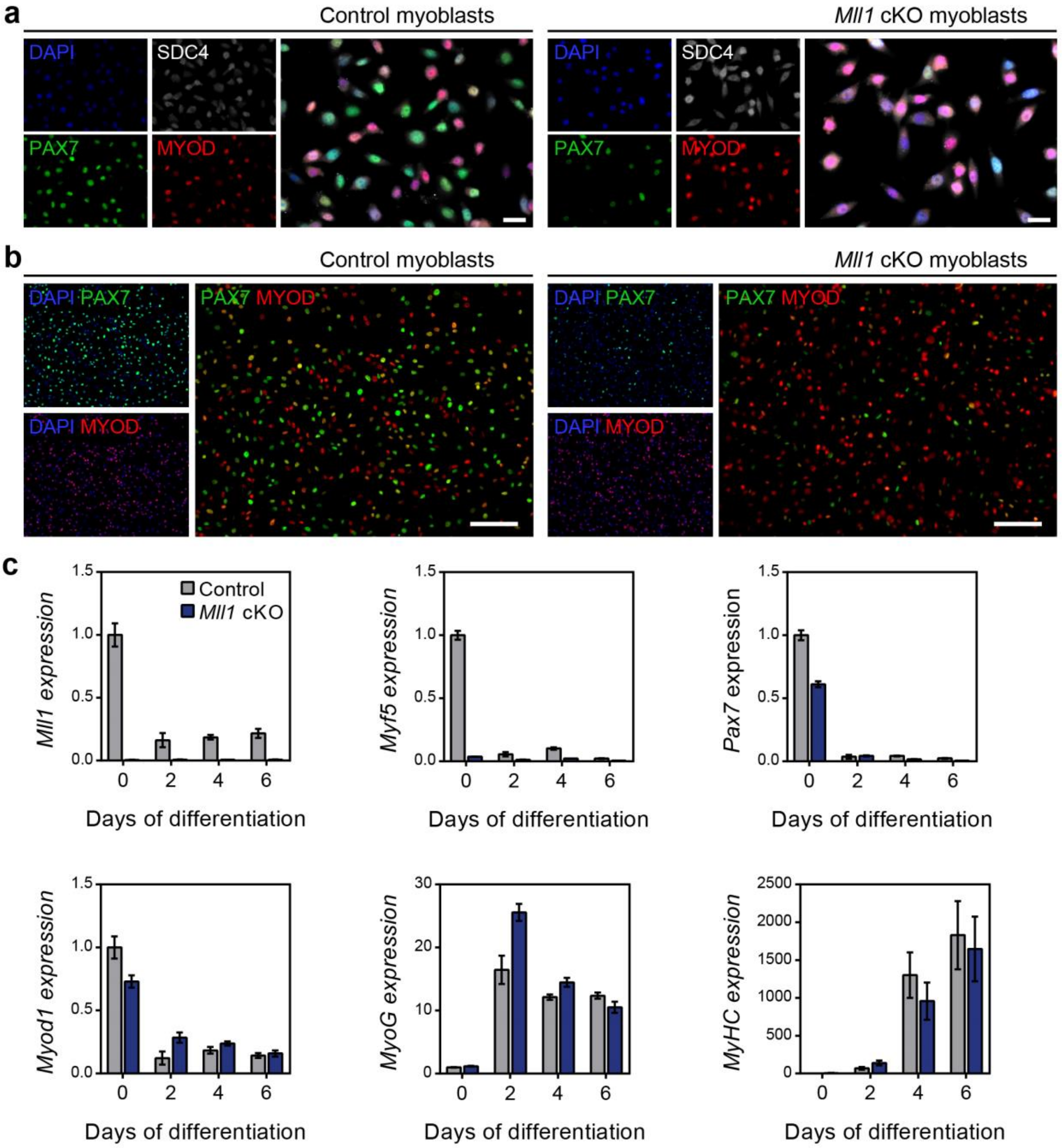

Supplementary Figure 3. Expression of *Mll1*, *Pax7* and myogenic markers in control and *Mll1* cKO primary myoblasts, during proliferation and myogenic differentiation.

(a) Immunostaining of PAX7 (green), MYOD (red) and SDC4 (white) in control and *Mll1* cKO proliferating myoblasts. Nuclei are counterstained with DAPI (blue). Scale bar represents 20µm.

(b) *Mll1* cKO proliferating myoblasts lose PAX7 expression (green), but maintain MYOD expression (red) compared to control myoblasts. Nuclei are counterstained with DAPI (blue). Scale bar represents 100µm.

(c) Control (grey bars) and *Mll1* cKO (blue bars) myoblasts were differentiated for 6 days and gene expression was analyzed every 2 days by RT-qPCR (normalized to *Hprt* expression). Data are presented as the mean ± S.E.M. of 3 independent experiments.

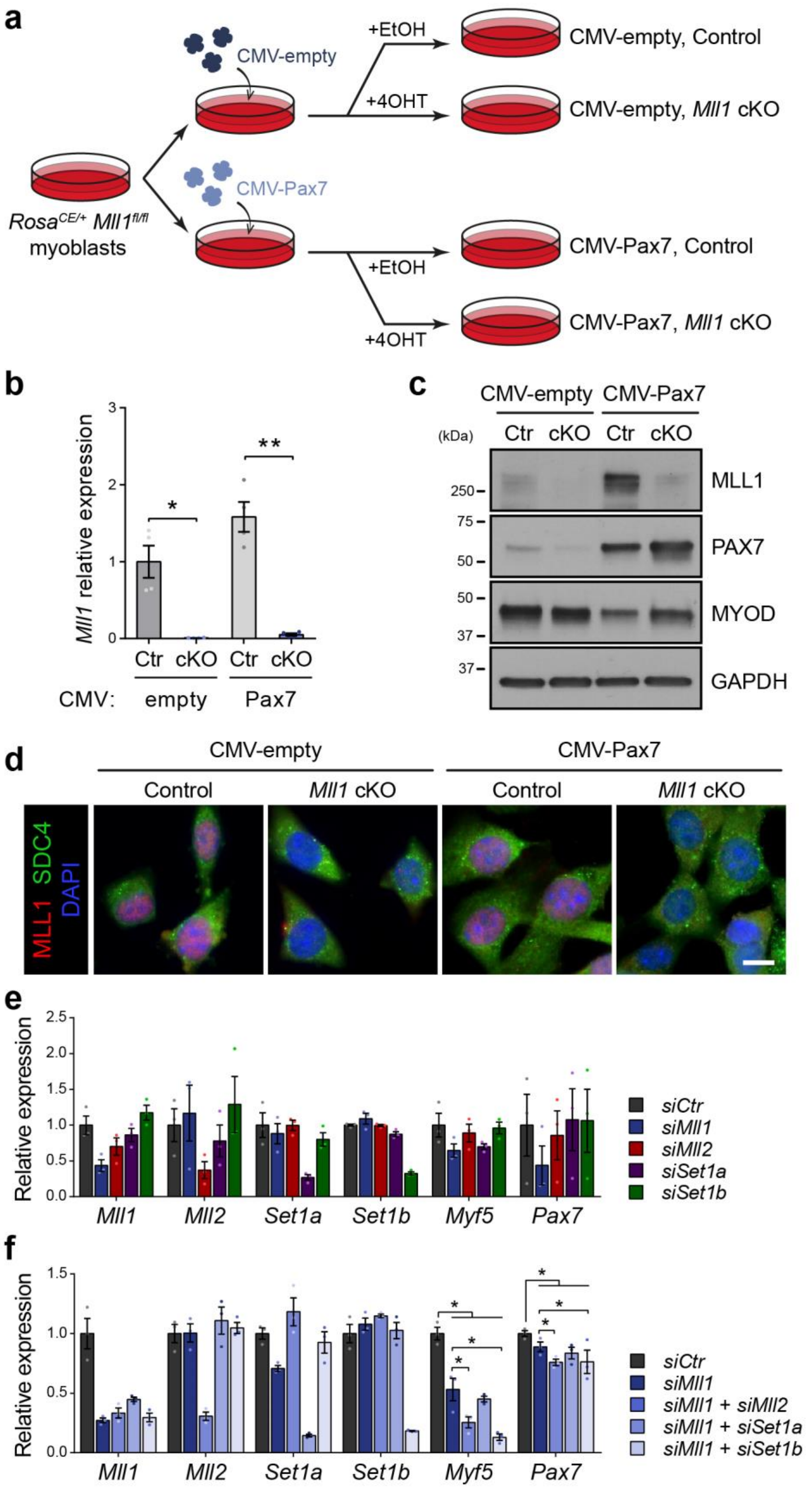

Supplementary Figure 4. Generation of *Mll1* cKO myoblasts overexpressing Pax7 and analysis of potential histone methyltransferase redundancy in regulating *Pax7* and *Myf5* expression.

(a) Experimental schematic outlining the protocol followed to obtain control and *Mll1* cKO primary myoblasts overexpressing, or not, *Pax7*. First, *Rosa*$^{CE/+}$:*Mll1*$^{fl/fl}$ myoblasts were transduced either with

an empty plasmid or a CMV-Pax7 plasmid. Then, transduced myoblasts were cultured for 7 days with either vehicle (ethanol 96%, EtOH), or 4-hydroxytamoxifen (4OHT) allowing for CreER translocation to the nucleus and subsequent Cre/LoxP-mediated recombination, to obtain control and *Mll1* cKO primary myoblasts, respectively.

(b) RT-qPCR analysis of *Mll1* mRNA expression (normalized to *Ppia* and *Rps18* expression) performed on control and *Mll1* cKO primary myoblasts transduced with either an empty or a CMV-Pax7 plasmid, represented as mean ± S.E.M. (n = 4 independent experiments).

(c) Western blot analysis of MLL1, PAX7 and MYOD in control and *Mll1* cKO primary myoblasts transduced with either an empty or a CMV-Pax7 plasmid. GAPDH is used as a loading control.

(d) Immunostaining of MLL1 (red) and SDC4 (green) in control and *Mll1* cKO proliferating myoblasts transduced with either an empty or a CMV-Pax7 plasmid. Nuclei are counterstained with DAPI (blue). Scale bar represents 10µm.

(e, f) Expression level of *Pax7* and *Myf5* in primary myoblasts, after transfection with siRNAs against *Mll1*, *Mll2*, *Set1a* and *Set1b*, individually (e) or in combination with *Mll1* siRNA (f), as indicated. Gene expression was determined by RT-qPCR (normalized to *Rps18* expression). Error bars represent SEM (n = 3 biological replicates).

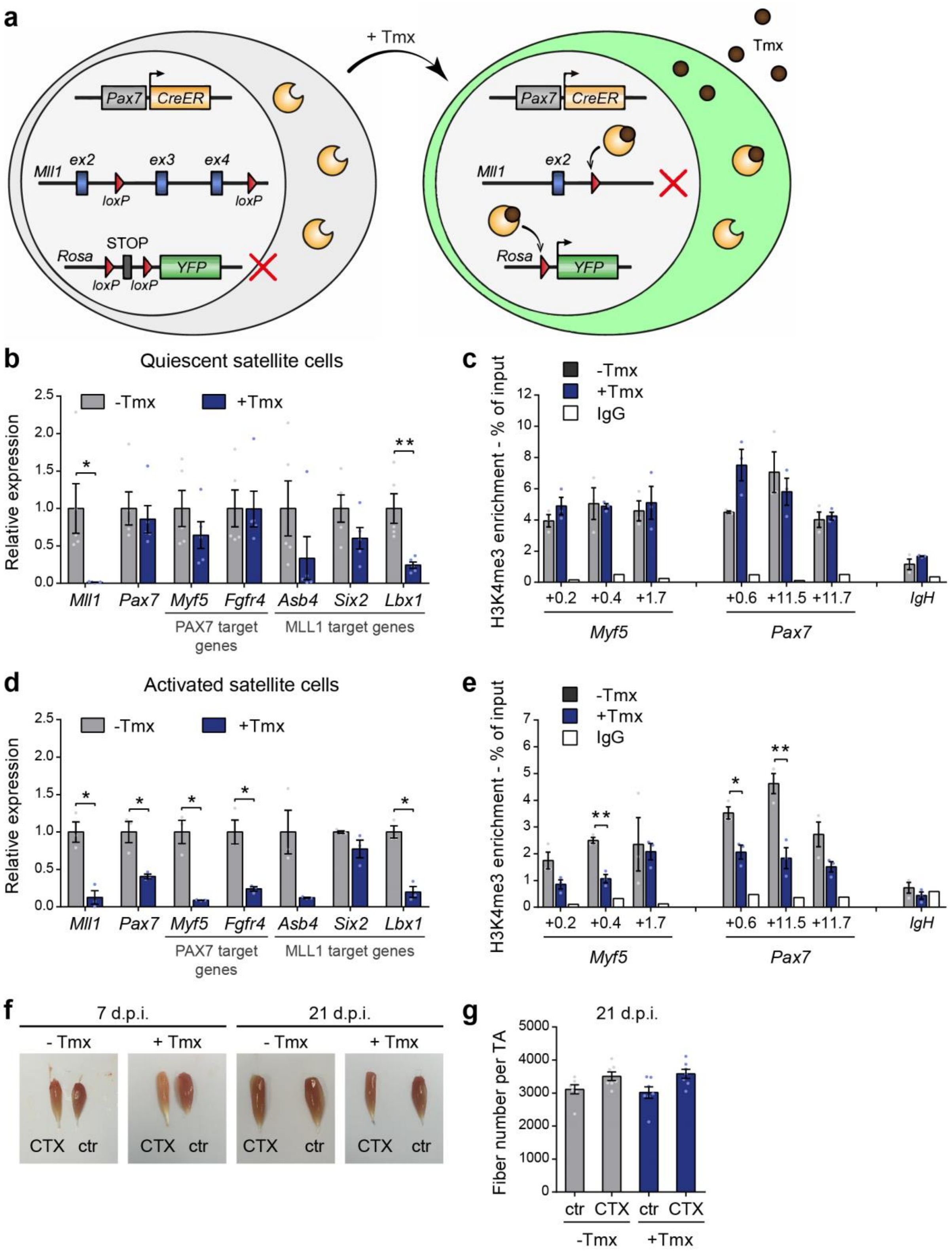

Supplementary Figure 5. Schematic model of *Pax7*$^{CE/+}$*:Mll1*$^{fl/fl}$*:Rosa*^{YFP} mice, model validation and phenotypic analysis.

(a) *Pax7*$^{CE/+}$*:Mll1*$^{fl/fl}$*:Rosa*^{YFP} mice generate CreER protein in all *Pax7*-expressing cells, however remaining in the cytoplasm in absence of tamoxifen (Tmx). Owing to the stop cassette in the modified Rosa gene, YFP is not expressed. Upon tamoxifen administration, tamoxifen binds to the CreER,

allowing for its translocation to the nucleus. Thus, tamoxifen-bound CreER recombine the *Mll1* locus, excising the exons 3 and 4 between the loxP sites, and the Rosa locus, excising the stop cassette and allowing for YFP expression. These PAX7-expressing cells are now labelled by the YFP but do not express MLL1.

(b) RT-qPCR analysis of *Mll1*, *Pax7* and their target genes in quiescent satellite cells, freshly isolated from $Pax7^{CE/+}$:$Mll1^{fl/fl}$:$Rosa^{YFP}$ mice treated with tamoxifen (+ Tmx), or not (- Tmx) (normalized to *Rps18* expression). Values represent mean ± S.E.M. (n = 5 mice per group).

(c) ChIP-qPCR analysis of H3K4me3 enrichment at *Myf5* and *Pax7* loci in quiescent satellite cells from $Pax7^{CE/+}$:$Mll1^{fl/fl}$:$Rosa^{YFP}$ mice treated with tamoxifen (+ Tmx), or not (- Tmx), represented as mean ± S.E.M. (n = 3 mice per group). *IgH* enhancer region is used as a negative control.

(d) RT-qPCR analysis of *Mll1*, *Pax7* and their target genes in activated satellite cells, freshly sorted 3 days after cardiotoxin (CTX)-induced injury from $Pax7^{CE/+}$:$Mll1^{fl/fl}$:$Rosa^{YFP}$ mice treated with tamoxifen (+ Tmx), or not (- Tmx) (normalized to *Rps18* expression). Values represent mean ± S.E.M. (n = 3 mice per group).

(e) ChIP-qPCR analysis of H3K4me3 enrichment at *Myf5* and *Pax7* loci in activated satellite cells from $Pax7^{CE/+}$:$Mll1^{fl/fl}$:$Rosa^{YFP}$ mice treated with tamoxifen (+ Tmx), or not (- Tmx), represented as mean ± S.E.M. (n = 3 mice per group). *IgH* enhancer region is used as a negative control.

(f) Representative photographs of contralateral (ctr) and CTX-injured *Tibialis anterior* (TA) muscles isolated from $Pax7^{CE/+}$:$Mll1^{fl/fl}$ mice treated or not with tamoxifen at 7 days or 21 days post-injury (d.p.i.).

(g) Fiber number of contralateral (ctr) and CTX-injured TA muscles from $Pax7^{CE/+}$:$Mll1^{fl/fl}$ mice treated or not with tamoxifen at 21 days post-injury (n = 7 mice per group).

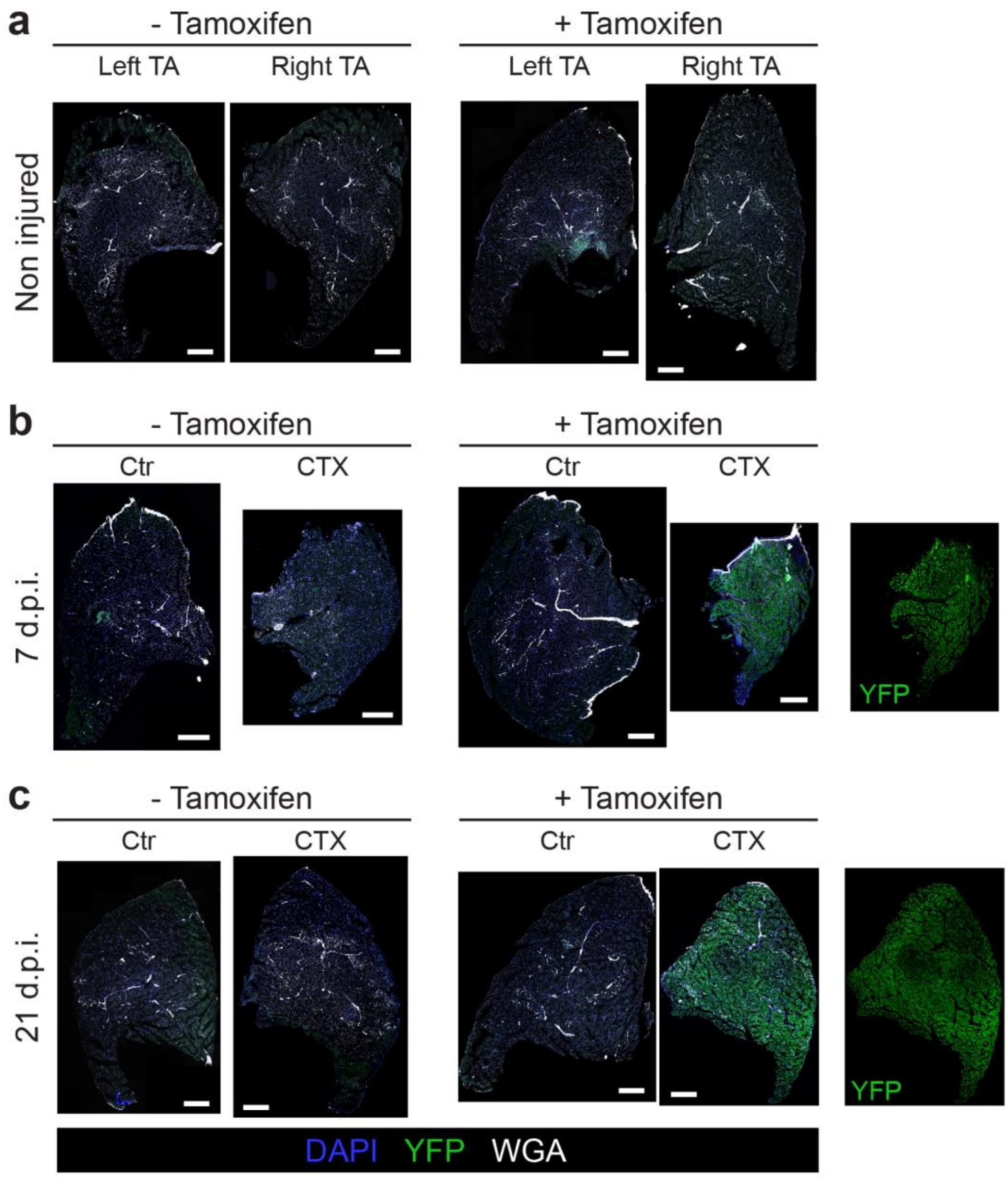

Supplementary Figure 6. Tracing YFP labeling during muscle regeneration of *Pax7$^{CE/+}$:Mll1$^{fl/fl}$:Rosa^{YFP}* mice.

YFP (green) immunostaining and wheat germ agglutinin (WGA, white) staining of (a) uninjured TA muscles, (b) CTX-injured and contralateral (ctr) TA muscles at 7 days post-injury (d.p.i.), and (c) CTX-injured and contralateral (ctr) TA muscles at 21 days post-injury (d.p.i.) from *Pax7$^{CE/+}$:Mll1$^{fl/fl}$* mice treated (+) or not (-) with tamoxifen. Nuclei are stained with DAPI (blue), and scale bars represent 500µm.

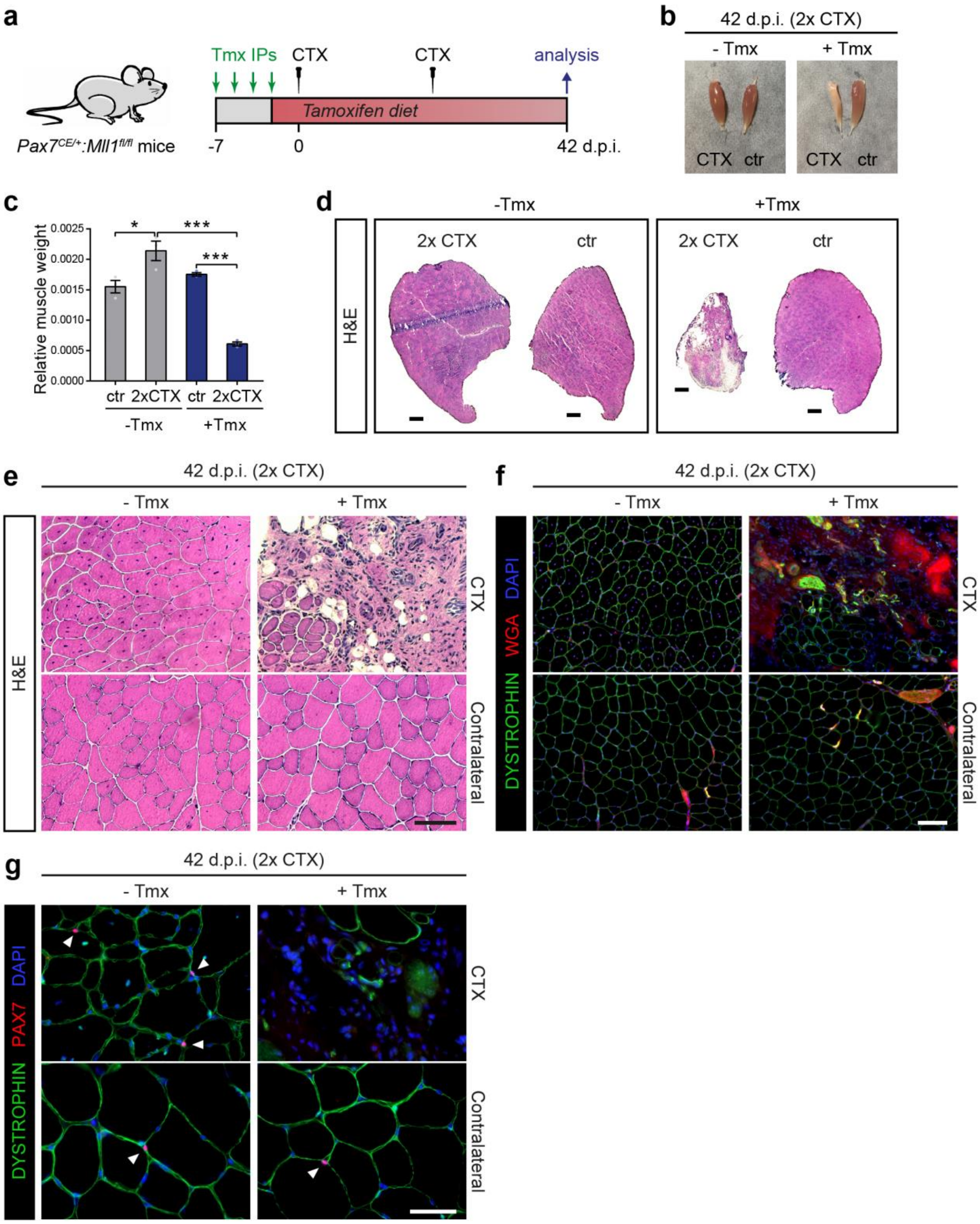

Supplementary Figure 7. Related to Figure 7. Tamoxifen-treated *Pax7*$^{CE/+}$*:Mll1*$^{fl/fl}$*:Rosa*^{YFP} mice do not regenerate after a second round of cardiotoxin injury.

(a) Experimental schematic outlining the protocol followed to analyzed muscle regeneration of *Pax7*$^{CE/+}$*:Mll1*$^{fl/fl}$ mice, following a double injury induced by cardiotoxin (CTX).

(b) Representative photographs of contralateral (ctr) and CTX-injured TA muscles isolated from *Pax7*$^{CE/+}$*:Mll1*$^{fl/fl}$ mice treated (+ Tmx) or not (- Tmx) with tamoxifen after two rounds of injuries (2x CTX) at 42 days post-injury (d.p.i.).

(c) Relative muscle weight of cardiotoxin double-injured (2x CTX) and contralateral (ctr) TA muscles normalized to the body weight at 42 days post-injury, represented as mean ± S.E.M (n = 3 mice per group).
(d) Representative hematoxylin and eosin (H&E) staining of the full cross-sections of double-injured (2x CTX) and contralateral (ctr) TA muscles from *Pax7*$^{CE/+}$*:Mll1*$^{fl/fl}$ mice, treated or not with tamoxifen at 42 days post-injury. Scale bars represent 500µm.
(e) H&E staining of double-injured and contralateral TA muscles from *Pax7*$^{CE/+}$*:Mll1*$^{fl/fl}$ mice, treated or not with tamoxifen at 42 days post-injury. Scale bar represents 100µm.
(f) Wheat germ agglutinin (WGA, red) and DYSTROPHIN (green) immunostaining of double-injured and contralateral TA muscles from *Pax7*$^{CE/+}$*:Mll1*$^{fl/fl}$ mice, treated or not with tamoxifen at 42 days post-injury. Nuclei are stained with DAPI (blue). Scale bar represents 100µm.
(g) PAX7 (red) immunostaining showing the absence of satellite cells following the second CTX injury in *Pax7*$^{CE/+}$*:Mll1*$^{fl/fl}$ mice, treated with tamoxifen. DYSTROPHIN (green) staining delineates the myofibers in green. Nuclei are counterstained with DAPI (blue). Scale bars represent 50µm.

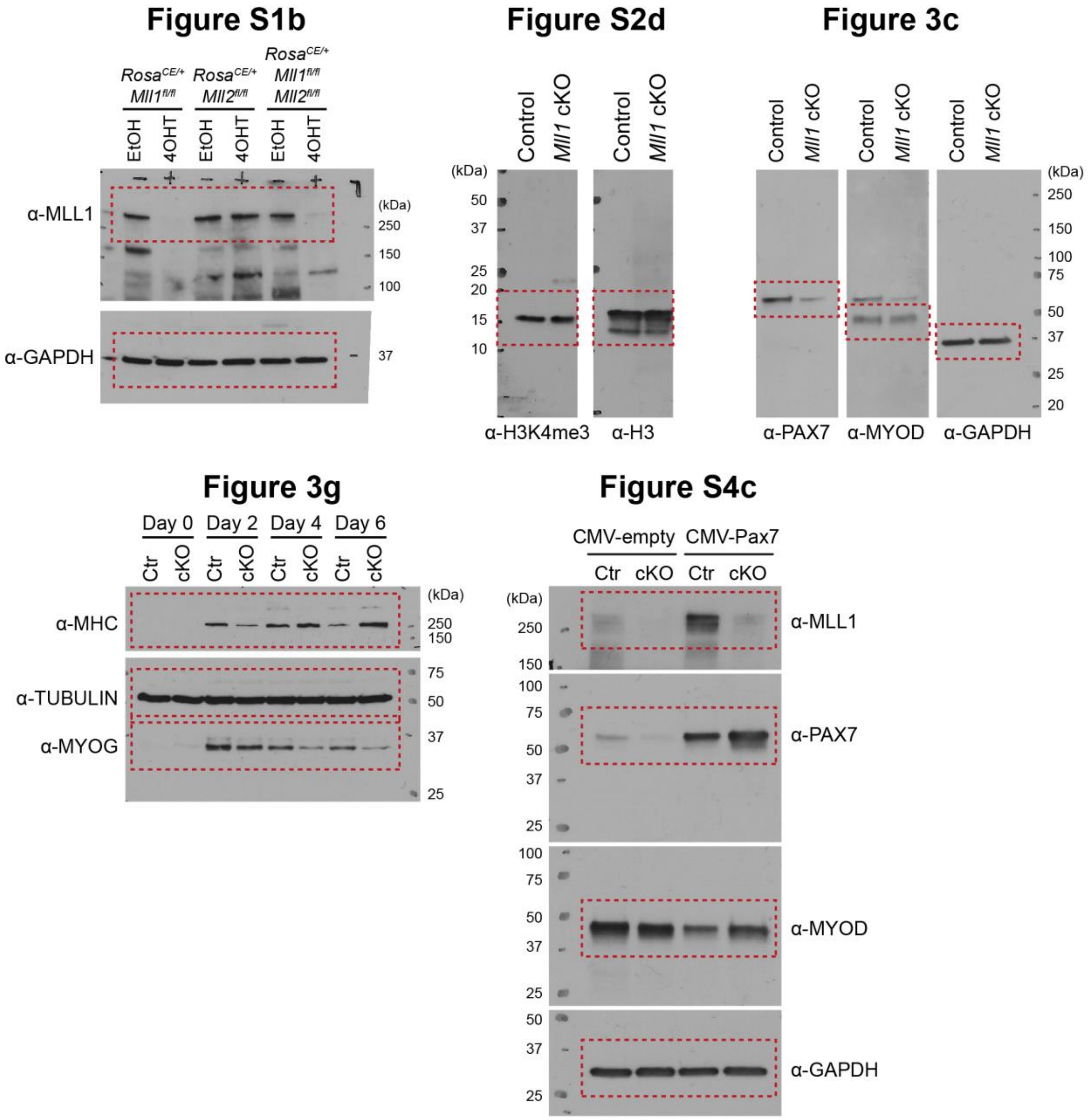

Supplementary Figure 8. Full scans of immunoblots.

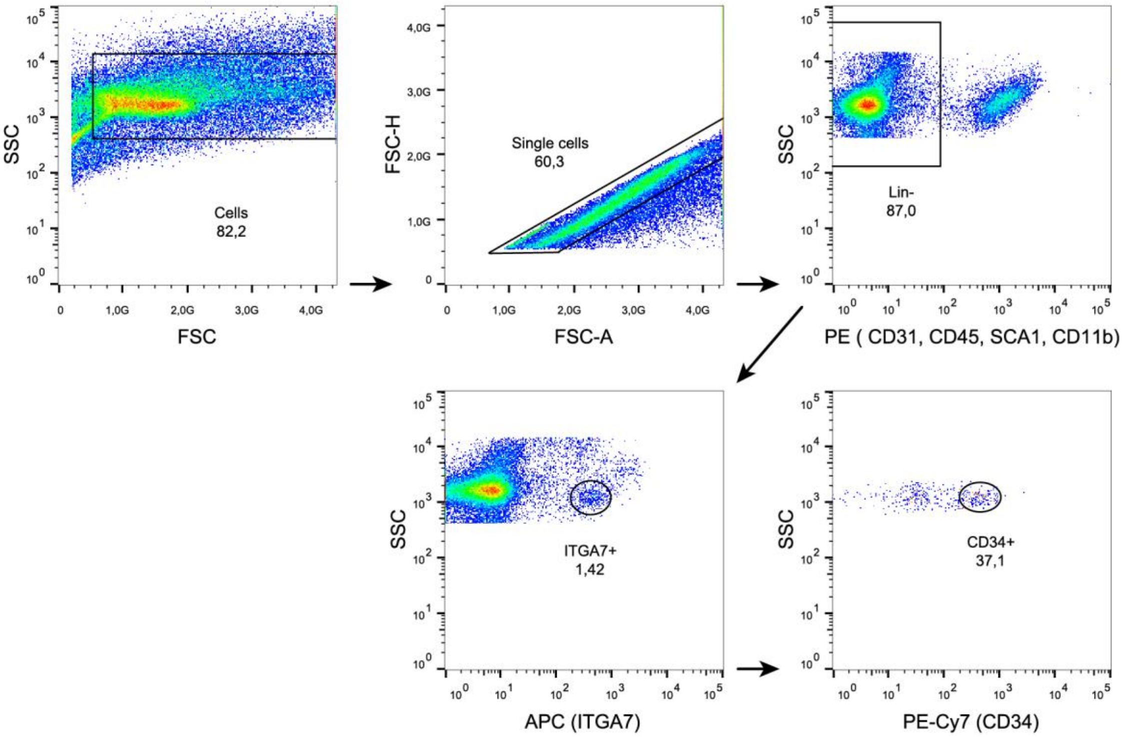

Supplementary Figure 9. Gating strategy for satellite cell sort.
Single cell suspensions were obtained from hindlimb muscles. Cell doublets were excluded from the analysis (FSC-H *vs* FSC-A). Satellite cells (CD31-, CD45-, SCA1-, CD11b-, Itga7+, CD34+) were sorted and used for analysis.

Supplementary Table 1. List of primers used for RT-qPCR analysis.

Gene	Forward	Reverse
Asb4	AGTTGTGGTCAATGCCTATG	AGCACACCTTGAAGAGAGA
Cd24a	GATGGTGGCCAGGCTAGGG	GGTGGTAGCGTTACTTGGATTTGGG
Dlk1	CAGCTACGACCTGGGCTACACC	CTGGAGTAGATGGTGCGTGGTTTCC
Dlx1	GTACTTAGCTCTGCCTGAGA	CCTTGCTTCATCAGCTTCTT
Fgfr4	GTGTCCACCACATTGACTAC	AAGACCACACGTCACTCT
Hprt	GGCCAGACTTTGTTGGATTTG	CACAGGACTAGAACACCTGC
Lbx1	TCGCCAGCAAGACCTTTA	ACTCGTAGATCTGGTGGTTG
MLL1	ATCGGCACCAACCTGCGC	TTTGGTCTCTGATTTGTTTAGAGGG
MLL2	TCCGAGGAGGAGGAGTTTCAGG	TCGTCTTATGCTTGCGGCCC
Myf5	CACCAACCCTAACCAGAGACTCCC	GCTGTTACATTCAGGCATGCCG
Myf6	GTCTACAGGACCTGCTGCACC	GCGCAGGAAATCCGCACCC
MyHC	GACCAGATCTTCCCCATGAA	TAAGGGTTGACGGTGACACA
Myod1	CATGATGGATTACAGCGGCCC	TCTGTGTCGCTTAGGGATGCCC
Myog	GCAATGCACTGGAGTTCG	ACGATGGACGTAAGGGAGTG
Pax7	CGACTCTGGATTCGTCTCC	GGCCTTGGCCAAGAGGG
Rps18	AACGGTCTAGACAACAAGCTG	AGTGGTCTTGGTGTGCTGAC
Sca1	GTGCAGCCCTTCTCTGAGGATGG	GTCTCAAATGGGACTCCATAGCACTGG
Set1a	TTGATGCCACCAAGTGTG	CTGCTTCGAGTAGATGACAATC
Set1b	GTAGACCATGACACCATCATC	ACGATCTTCTTCTGGGACT
Sfrp2	GCCTTCGGCTTCCCCTGG	GCAGGCTTCACACACCTTGGG
Six2	CAAGTCAGCAACTGGTTCA	TGGAGTTCTCGCTGTTCT

Supplementary Table 2. List of primers used for ChIP-qPCR experiments.

Locus	Forward	Reverse
Pax7 -27	GCTTGCAGAGAGACTTGCC	CAGTATAGACCAGGCTGGCC
Pax7 -0.2	CGCAAGGCGCAGCTGGG	GGGAGGAGTGGAAGGTGG
Pax7 +0.6	GTCCCTCAAAGTCGGGATGG	GCGGGAAATAAGGCACTATGC
Pax7 +1.1	GGGTGCGGGAGGATGGG	TTCGCCTCTGTCTAATTCGGG
Pax7 +11.5	AAATCTAATCAAGGCAGAACGCG	CTTGGAATCCCGGTCTCGC
Pax7 +11.7	GCCAAACGAAAGGATGTGGG	CGCTTGGAAACTGGATTTGGG
IgH	GCCGATCAGAACCAGAACAACTGC	TGGTGGGGCTGGACAGAGTGTTTC
Myf5 -6.4	GATGCAATCCTCCTGCGTGG	AAGACTGCTGGAGGCTGAGG
Myf5 -3.5	CAAAATATACGTAACTCAGTCTGGG	CATATCAATTGCACACAGAATCTGG
Myf5 +0.2	CCTGCTTCTCTGAAGGATGG	CGAAGGCTGCTACTCTTGGC
Myf5 +0.4	CCAGGCTGGCCACTGCC	CTTGGTTGACCTTCTTCAGGC
Myf5 +1.1	GGGCGCATTGCTGCGGG	CAGCACTGATCTAGGAAGGG
Myf5 +1.7	CTTTTGCTTGAAATATTACCAGGG	CTTGTGGTGCCCTTTAAACCC
Myf5 +4.9	CTGTTTACCCAATAGCCTACC	TATGCTGCCTTGCCTGCCC

Supplementary Table 3. List of antibodies and reagents.

Primary Antibody	Company	Reference	Dilution WB	Dilution IF/Flow
Mouse anti-PAX7	DSHB (Hybridoma)	PAX7	1:10	1:2
Mouse anti-TUBULIN (clone DM1A)	Sigma	T9026	1:5000	N/A
Mouse anti-GAPDH	AbLab	21-0017	1:5000	N/A
Mouse anti-Myosin Heavy Chain	DSHB (Hybridoma)	MF20	1:1000	1:500
Mouse anti-MYH3 (embryonic Myosin)	DSHB	F1.652	N/A	1:100
Rabbit anti-MYOD (C-20)	Santa Cruz	sc-304	N/A	1:1000
Mouse anti-MYOD (5.8A)	Dako	M3512	1:2000	N/A
Mouse anti-MYOGENIN (clone F5D)	Santa Cruz	sc-12732	1:1000	N/A
Rabbit anti-DYSTROPHIN	Abcam	ab15277	N/A	1:1000
Chicken anti-SYNDECAN-4	Gift from Dr. B. Olwin	N/A	N/A	1:1000
Chicken anti-GFP	Abcam	ab13970	N/A	1:500
Rabbit anti-Ki67	Abcam	ab15580	N/A	1:1000
Rabbit anti-H3K4me3	Abcam	ab8580	1:1000	N/A
Rabbit anti-H3K4me3	Millipore	07-473	N/A	N/A
Rabbit anti-H3	Abcam	ab1791	1:2500	N/A
Normal Rabbit IgG	Millipore	12-370	N/A	N/A
Normal Mouse IgG	Millipore	12-371	N/A	N/A
Rabbit anti-Mouse IgG	Jackson Labs	315-005-008	N/A	N/A
Mouse anti-MLL1	Millipore	05-764	1:500	1:250
Alexa647 mouse anti-Integrin alpha7 (clone R2F2)	UBC AbLab	67-0010-10	N/A	1:100
Mouse anti-CD34-Biotin (clone REA383)	Miltenyi Biotec	130-105-830	N/A	1:100
PE mouse anti-SCA1 (clone D7)	BD Biosciences	553108	N/A	1:500
PE mouse anti-CD45 (clone 30-F11)	BD Biosciences	12-0451-83	N/A	1:500
PE mouse anti-CD31 (clone 390)	BD Biosciences	12-0311-81	N/A	1:500
PE mouse anti-CD11b (clone M1/70)	BD Biosciences	12-0112-81	N/A	1:500
**Reagent**	**Company**	**Reference**	**Dilution WB**	**Dilution IF/Flow**
Streptavidin PE-Cyanine7 Conjugate	ThermoFisher Scientific	25-4317-82	N/A	1:500
**Secondary Antibody**	**Company**	**Reference**	**Dilution WB**	**Dilution IF/Flow**
Goat anti-Mouse IgG1, Alexa Fluor 546	ThermoFisher Scientific	A-21123	N/A	1:1000
Goat anti-Chicken IgY, Alexa Fluor 488	ThermoFisher Scientific	A-11039	N/A	1:1000
Goat anti-Chicken IgY, Alexa Fluor 647	ThermoFisher Scientific	A-21449	N/A	1:1000
Goat anti-Rabbit IgG, Alexa Fluor 488	ThermoFisher Scientific	A-11034	N/A	1:1000
**Lectin**	**Company**	**Reference**	**Dilution WB**	**Dilution IF/Flow**
Wheat Germ Agglutinin, Alexa Fluor 647 Conjugate	ThermoFisher Scientific	W32466	N/A	1:500
