# Supplementary material for: MLL1 is required for PAX7 expression and satellite cell self-renewal in mice
Source: Nat Commun. 2019 Sep 18;10:4256. doi: 10.1038/s41467-019-12086-9 (PMC6751293; doi:10.1038/s41467-019-12086-9)
Supplement: Supplementary file 3 — Description of Additional Supplementary Files [file 41467_2019_12086_MOESM3_ESM.pdf]

Description of Additional Supplementary Files

File Name: Supplementary Data file 1.

Description: Microarray data from control and Mll1 cKO myoblasts.

File Name: Supplementary Data file 2.

Description: GO term analysis of the up-regulated genes in Mll1 cKO myoblasts.
