# Supplementary material for: MLL1 is required for PAX7 expression and satellite cell self-renewal in mice
Source: Nat Commun. 2019 Sep 18;10:4256. doi: 10.1038/s41467-019-12086-9 (PMC6751293; doi:10.1038/s41467-019-12086-9)
Supplement: Supplementary file 6 — Reporting Summary [file 41467_2019_12086_MOESM6_ESM.pdf]

nature research

Corresponding author(s): Michael A. Rudnicki

Last updated by author(s): Jul 19, 2019

Reporting Summary

Nature Research wishes to improve the reproducibility of the work that we publish. This form provides structure for consistency and transparency in reporting. For further information on Nature Research policies, see Authors & Referees and the Editorial Policy Checklist.

Statistics

For all statistical analyses, confirm that the following items are present in the figure legend, table legend, main text, or Methods section.

n/a	Confirmed	
☐	☒	The exact sample size (n) for each experimental group/condition, given as a discrete number and unit of measurement
☐	☒	A statement on whether measurements were taken from distinct samples or whether the same sample was measured repeatedly
☐	☒	The statistical test(s) used AND whether they are one- or two-sided<br>*Only common tests should be described solely by name; describe more complex techniques in the Methods section.*
☒	☐	A description of all covariates tested
☒	☐	A description of any assumptions or corrections, such as tests of normality and adjustment for multiple comparisons
☒	☐	A full description of the statistical parameters including central tendency (e.g. means) or other basic estimates (e.g. regression coefficient) AND variation (e.g. standard deviation) or associated estimates of uncertainty (e.g. confidence intervals)
☐	☒	For null hypothesis testing, the test statistic (e.g. F, t, r) with confidence intervals, effect sizes, degrees of freedom and P value noted<br>*Give P values as exact values whenever suitable.*
☒	☐	For Bayesian analysis, information on the choice of priors and Markov chain Monte Carlo settings
☒	☐	For hierarchical and complex designs, identification of the appropriate level for tests and full reporting of outcomes
☒	☐	Estimates of effect sizes (e.g. Cohen's d, Pearson's r), indicating how they were calculated

Our web collection on statistics for biologists contains articles on many of the points above.

Software and code

Policy information about availability of computer code

Data collection	Bio-Rad CFX96 Maestro instrument (qPCR), Zeiss Axio Observer.D1 (Microscopy), Zeiss Axio Observer.A1 (Microscopy), Summit (FACS)
Data analysis	Microarrays: R (Bioconductor package), GraphPad Prism v7.00, MATLAB-MathWorks r2015a, MATLAB application SMASH (Semi-Automatic Muscle Analysis using Segmentation of Histology), Zeiss ZEN 2 software (Microscopy), Image J FIJI (imaging), FlowJo v10

For manuscripts utilizing custom algorithms or software that are central to the research but not yet described in published literature, software must be made available to editors/reviewers. We strongly encourage code deposition in a community repository (e.g. GitHub). See the Nature Research guidelines for submitting code & software for further information.

Data

Policy information about availability of data

All manuscripts must include a data availability statement. This statement should provide the following information, where applicable:

- Accession codes, unique identifiers, or web links for publicly available datasets
- A list of figures that have associated raw data
- A description of any restrictions on data availability

Microarray data are available on NCBI GEO accession number [GSE108339] (https://www.ncbi.nlm.nih.gov/geo/).
The datasets generated during and/or analysed during the current study are available from the corresponding author on reasonable request.

Field-specific reporting

Please select the one below that is the best fit for your research. If you are not sure, read the appropriate sections before making your selection.

☒ Life sciences ☐ Behavioural & social sciences ☐ Ecological, evolutionary & environmental sciences

For a reference copy of the document with all sections, see nature.com/documents/nr-reporting-summary-flat.pdf

Life sciences study design

All studies must disclose on these points even when the disclosure is negative.

Sample size	Sample size is indicated in the figure legends for each experiment. No sample size calculation was performed. Sample size was determined based on the magnitude and consistency of measurable differences between groups.
Data exclusions	No data were excluded from the analysis.
Replication	Biological and independent replicate experiments were successful. They were replicated independently by the different co-authors.
Randomization	The sex and age-matched mice were randomized into control and tamoxifen-treated groups.
Blinding	The researchers were blinded to allocation during analysis and outcome assessment. Animal experiments were blinded when possible.

Reporting for specific materials, systems and methods

We require information from authors about some types of materials, experimental systems and methods used in many studies. Here, indicate whether each material, system or method listed is relevant to your study. If you are not sure if a list item applies to your research, read the appropriate section before selecting a response.

Materials & experimental systems

n/a	Involved in the study	
☐	☒	Antibodies
☒	☐	Eukaryotic cell lines
☒	☐	Palaeontology
☐	☒	Animals and other organisms
☒	☐	Human research participants
☒	☐	Clinical data

Methods

n/a	Involved in the study	
☒	☐	ChIP-seq
☐	☒	Flow cytometry
☒	☐	MRI-based neuroimaging

Antibodies

Antibodies used	The list of antibodies used can be find in the manuscript, Supplementary Table 3.
Validation	All the antibodies were validated in previous published work from the lab and by the manufacturers listed in the Supplementary Table 3.

Animals and other organisms

Policy information about studies involving animals; ARRIVE guidelines recommended for reporting animal research

Laboratory animals	Mixed genetic background (129SV and C57BL/6) mice were used in the study. Information about genotype, breeding, sex and age can be found in the "Methods" section of the manuscript (Mice and Animal care).
Wild animals	Study did not involve wild animals.
Field-collected samples	Study did not involve field-collected samples..
Ethics oversight	All experimental protocols for mice used in this study were performed in accordance with the guidelines established by the University of Ottawa Animal Care Committee, which is based on the guidelines of the Canadian Council on Animal Care (CCAC). Protocols were approved by the Animal Research Ethics Board (AREB) at the University of Ottawa.

Note that full information on the approval of the study protocol must also be provided in the manuscript.

Flow Cytometry

Plots

Confirm that:

☒ The axis labels state the marker and fluorochrome used (e.g. CD4-FITC).

☒ The axis scales are clearly visible. Include numbers along axes only for bottom left plot of group (a 'group' is an analysis of identical markers).

☒ All plots are contour plots with outliers or pseudocolor plots.

☒ A numerical value for number of cells or percentage (with statistics) is provided.

Methodology

Sample preparation	Sample preparation for fluorescent-activated cell sorting can be found in the "Methods" section of the manuscript (Fluorescence-activated cell sorting).
Instrument	FACS was performed on MoFlo XDP at the Ottawa Hospital Research Institute.
Software	Data acquisition: Summit, Data analysis:FlowJo
Cell population abundance	Purity of the post-sort fractions was determined by flow cytometry on the sorted samples. Only samples that were >90% pure were kept for analysis.
Gating strategy	Gating strategy was performed using unstained and single-stained samples. The gating strategy for sorting the satellite cells is provided in Supplementary Figure 9.

☒ Tick this box to confirm that a figure exemplifying the gating strategy is provided in the Supplementary Information.
